# Pancreatic Lineage Specifier PDX1 Increases Adhesion and Decreases Motility of Cancer Cells

**DOI:** 10.3390/cancers13174390

**Published:** 2021-08-30

**Authors:** Liya Kondratyeva, Igor Chernov, Eugene Kopantzev, Dmitry Didych, Alexey Kuzmich, Irina Alekseenko, Sergey Kostrov, Eugene Sverdlov

**Affiliations:** 1 Shemyakin-Ovchinnikov Institute of Bioorganic Chemistry of the Russian Academy of Sciences, Ulitsa Miklukho-Maklaya, 117997 Moscow, Russia; igor.palich@gmail.com (I.C.); kopantzev@gmail.com (E.K.); dmitrydid@gmail.com (D.D.); akrubik@gmail.com (A.K.); irina.alekseenko@mail.ru (I.A.); 2 Institute of Molecular Genetics of National Research Centre “Kurchatov Institute”, Ploshchad’ Akademika Kurchatova, 123182 Moscow, Russia; kostrov@img.ras.ru

**Keywords:** pancreatic cancer, PDX1, epithelial-mesenchymal transition, cancer metastasis, cell migration, cell adhesion

## Abstract

**Simple Summary:**

A possible way to inhibit tumor cells migration could be strengthening adhesion between tumor and stromal cells in primary solid tumors. Expanding on our previous findings, we have shown that ectopic expression of the pancreatic lineage specifier PDX1 reduces the migration potential of pancreatic cancer cells by increasing their adhesiveness and reducing the sensitivity to TGFβ1-induced epithelial-mesenchymal transition. We observed decreased expression levels of genes associated with promoting cell migration and increased expression of genes negatively affecting cell motility. Our study highlights a potential window for the PDX1 therapeutic application as an antimetastatic agent at the initial stages of cancer development.

**Abstract:**

Intercellular interactions involving adhesion factors are key operators in cancer progression. In particular, these factors are responsible for facilitating cell migration and metastasis. Strengthening of adhesion between tumor cells and surrounding cells or extracellular matrix (ECM), may provide a way to inhibit tumor cell migration. Recently, we demonstrated that PDX1 ectopic expression results in the reduction of pancreatic cancer line PANC-1 cell motility in vitro and in vivo, and we now provide experimental data confirming the hypothesis that suppression of migration may be related to the effect of PDX1 on cell adhesion. Cell migration analyses demonstrated decreased motility of pancreatic Colo357 and PANC-1 cell lines expressing PDX1. We observed decreased expression levels of genes associated with promoting cell migration and increased expression of genes negatively affecting cell motility. Expression of the EMT regulator genes was only mildly induced in cells expressing PDX1 during the simulation of the epithelial-mesenchymal transition (EMT) by the addition of TGFβ1 to the medium. PDX1-expressing cancer cell lines showed increased cell adhesion to collagen type I, fibronectin, and poly-lysine. We conclude that ectopic expression of PDX1 reduces the migration potential of cancer cells, by increasing the adhesive properties of cells and reducing the sensitivity to TGFβ1-induced EMT.

## 1. Introduction

Metastasis is thought to be the final step in the chain of evolutionary events during which numerous interactions between cancer cells and their microenvironment allow tumor cells to populate new tissue habitats. Cell survival in the new niche is promoted by the tumor microenvironment, which stimulates cell proliferation [1]. Metastases are responsible for 90% of cancer-related mortality [2]. A detailed study of the mechanisms of metastasis would allow inhibition or prevention of metastasis, thus reducing mortality in cancer patients.

In the classical view, during the first stages of malignant transformation, epithelial cells lose their dependence on integrin-mediated interactions with the extracellular matrix (ECM). During this process, intercellular contacts mediated by E-cadherin and interactions fundamental to epithelial cells are destroyed. The cells lose polarity, which leads to an increase in migratory and invasive capabilities [3,4]. These changes are associated with epithelial-mesenchymal transition (EMT), which makes cancer cells more mobile and invasive [5].

As an approach to slow down the formation of metastases, it has been proposed to enhance adhesion between tumor cells and surrounding cells and ECM in their primary localization [6]. However, attempts to use biochemical agents for this purpose have not yielded significant results [3]. Modulation of natural cellular mechanisms to increase the adhesive properties of cells may be more promising.

In our earlier work [7], we showed that expression of the embryonic pancreas lineage-specific transcription factor, pancreatic and duodenal homeobox 1 (PDX1), in the pancreatic cancer cell line, PANC-1, leads to a decrease in motility of cells and suppression of their ability to spread throughout the body in the model organism *Danio rerio*. Based on our results, we hypothesized that the decrease in cell migration capability may be due to the effect of PDX1 on the cells’ adhesion within their microenvironment.

PDX1 is an important regulatory transcription factor both for embryonic development of the pancreas as a whole and for differentiation of progenitor cells into Langerhans islet beta cells [8,9]. In the adult organism, PDX1 plays a key role in the maintenance and functioning of beta cells [10], PDX1 regulates the transcription of genes of insulin, somatostatin, glucagon, and islet amyloid polypeptide hormones as well as genes whose transcription depends on glucose concentration, such as β-glucokinase, *Kir 6.1*, and *Glut2* [11]. PDX1 expression is normally observed mainly in the endocrine part of the pancreas, in Langerhans islets cells, and in centroacinar cells [12]. A simultaneous significant increase in *PDX1* expression in tumor and adjacent tissues was reported for breast, prostate, colorectal, and renal cancers [13], whereas *PDX1* expression was not detected in corresponding healthy tissues. *PDX1* overexpression has also been observed in insulinoma, neuroendocrine tumors, and in some cases of pancreatic cancer [14]. During the course of pancreatic oncogenesis, PDX1 functions change. PDX1 first acts as a tumor suppressor, maintaining the identity of acinar cells. After the acquisition of the KRAS oncogenic mutation, it plays an oncogenic role, stimulating cell proliferation and inhibiting apoptosis. At later stages, it gains oncosuppressor properties possibly due to EMT inhibition [15,16]. The most recent evidence [17] suggests a possible modulation of the oncogenic/tumor-suppressive properties of PDX1 by non-receptor tyrosine kinase BLK activity.

PDX1 affects cell adhesion capability during embryonic development of the pancreas It binds to two conserved sites in the E-cadherin gene promoter and activates its transcription in mouse pancreatic precursor cells [18]. In addition, PDX1 is required for the maintenance of E-cadherin expression, actomyosin complex activity, and cell shape [18]. These data demonstrate a new relationship between regulators of epithelial architecture and regulators of differentiation programs for pancreatic precursor cells.

In this study, b175ased on previously obtained data [7], we studied the effect of *PDX1* expression on the proliferative and migratory capabilities of four pancreatic cancer cell lines (BxPC-3, Colo357, MiaPaCa-2, and PANC-1) and the colon cancer line SW620 in vitro. Cell motility-related gene expression, cell adhesion analysis, and sensitivity to EMT induction tests were performed for the most motile cells. The data obtained suggest that ectopic expression of PDX1 reduces the migration potential of cancer cells, in particular, by increasing the adhesive properties of cells and reducing the sensitivity of cells to TGFβ1-induced EMT.

## 2. Materials and Methods

### 2.1. Cell Cultures

PANC-1 (ATCC^®^ CRL-1469), MiaPaCa-2 (ATCC^®^ CRL-1420), BxPC-3 (ATCC^®^ CRL-1687), SW620 (ATCC^®^ CCL-227), and 293T (ATCC^®^ CRL-3216™) cells were obtained from the American Type Culture Collection (ATCC, Manassas, VA, USA). The authenticity of PANC-1, MiaPaCa-2, BxPC-3, 293T human cell lines used in this study has been proven by DNA profiling (OOO “Gordiz”, Moscow, Russia). Colo-357 (Proper Citation: (ECACC 94072245, CVCL_0221) cell line is a gift from R. Metzgar (Duke University, Durham, NC, USA). All experiments were performed with mycoplasma-free cells.

PANC-1, MiaPaCa-2, and 293T cells were cultured in Dulbecco′s modified Eagle′s medium (DMEM) supplemented with 100 U/mL penicillin, 100 mkg/mL streptomycin, and 10% fetal bovine serum. Colo357, BxPC-3, and SW620 cells were cultured in RPMI-1640 medium supplemented with 100 U/mL penicillin, 100 mkg/mL streptomycin, and 10% fetal bovine serum. Media and supplements were purchased from Gibco (Carlsbad, CA, USA). Cells were maintained in a humidified atmosphere containing 5% CO_2_ at 37 °C.

TGF-β-responsive control and PDX1-expressing Colo357 and PANC-1 cells with activating mutations of the K-Ras oncogene were tested in EMT-modeling experiments. When cells grew to 70% confluence, the routine medium was removed and replaced with serum-free RPMI 1640 or DMEM without or with 10 ng/mL TGFβ1 (Sigma-Aldrich, St. Louis, MO, USA) for 24 h at 37 °C. Cell shape parameter (height and width) of control and PDX1-expressing cells after TGFβ1 treatment was estimated using the free ImageJ software (version 1.50i, NIH, Bethesda, MD, USA). For each cell type, 45–50 individual cells were analyzed.

### 2.2. Lentivirus Vector Design, Production and Transduction of Cancer Cells

Three lentivirus vectors were designed to express GFP under the control of the CMV promoter, to express the PDX1 gene under the control of the PCNA promoter, and the puromycin-resistance gene under the control of the PGK promoter, and control vector to express solely the puromycin-resistance gene as previously described [7]. Obtained vectors containing PDX1 and control vector were transfected with Lipofectamine^®^ 2000 (Invitrogen Life Technologies, Carlsbad, CA, USA) into 80% confluent 293T cells for 48 h at 37 °C. The cell culture medium containing the lentivirus was collected and stored at 70 °C.

Colo357, MiaPaCa-2, BxPC-3, and SW620 cells were transduced with lentiviral particles containing the PCNA promoter to express PDX1 and those containing the control vector. Transduction was performed in 6-well plates seeded with 3 × 10^5^ cells/well followed by puromycin (Sigma-Aldrich, St. Louis, MO, USA) selection (4 μg/mL) for 10 days. The cell lines stably expressing PDX1 and control cells were maintained in puromycin (2 μg/mL). The generated stable cell lines were named [cell line name]-PDX1 and [cell line name]-Control, respectively. PANC-1-PDX1 and PANC-1-Control cells were obtained previously [7]. Next, PDX1-expressing and control cells were transduced with lentiviral particles containing Turbo-GFP under the control of the CMV promoter. The transduction was performed as described previously. GFP-positive (GFP+) transduced cells were selected using a fluorescence-activated cell sorter FACSAria III (BD Bioscience, San Jose, CA, USA). PANC-1-PDX1 and PANC-1-Control cells were obtained previously [7].

### 2.3. Western Blotting

The lentivirus-transduced cells were lysed in an SDS sample buffer containing 1% SDS, 2% 2-mercaptoethanol, and 62 mM Tris–HCl, pH 6.8, subjected to SDS electrophoresis on 10–15% polyacrylamide gels and then electrotransferred to a Polyvinylidene difluoride (PVDF) Immobilon-P membrane (Millipore, Burlington, MA, USA) using a Bio-Rad Trans-Blot SD cell (Bio-Rad Laboratories, Hercules, CA, USA). The membranes were blocked with 5% skimmed milk in PBS-T (PBS containing 0.1% Tween 20) for 1 h at room temperature, incubated in PBS-T containing 5% skimmed milk and the relevant primary antibody overnight at 4 °C, and finally washed three times with PBS-T. The membranes were then incubated with indicated primary antibodies rabbit anti-PDX1 (1:1000; Cat. no. 5679; Cell Signaling Technology, Danvers, MA, USA) and mouse anti-GAPDH (1:60,000; Cat. no. 10494–1-AP; Santa Cruz Biotechnology Inc., Dallas, TX, USA) at 4 °C overnight, followed by incubation with horseradish peroxidase (HRP)-conjugated secondary antibodies (1:5000; Cat. no. sc-2054; Santa Cruz Biotechnology Inc.) at room temperature for 1 h. Mouse monoclonal anti-GAPDH antibody was used as a loading control. After washing, the membranes were incubated in PBS-T containing 5% skimmed milk and goat anti-mouse or anti-rabbit antibody HRP conjugates (Santa Cruz, 1:5000) for 1 h at room temperature. The membranes were finally washed with PBS-T, and specific signals were visualized using a Clarity Western ECL (Bio-Rad Laboratories) with a VersaDoc Imaging System (Bio-Rad Laboratories). The densitometry analysis was performed with a VersaDoc Imaging System for quantification of relative PDX1 level. The values were calculated relative to the level of PDX1 in Colo357-PDX1 cells.

### 2.4. Cell Proliferation Assay

Following the lentivirus transduction, the PDX1-expressing and control cells (2000 cells/well) cells were seeded each into 96-well plates. The increase in the number of control and PDX1-expressing cells resulting from divisions was estimated by MTS assay during 7 days. MTS assay was performed using CellTiter 96^®^ AQueous One Solution Cell Proliferation Assay (MTS) (Promega, Madison, WI, USA). MTS solution (5 mg/mL; 20 µL) was added to each well and incubated for 1 h at 37 °C. The absorbance of each plate was measured at 595 nm using a Benchmark Plus microplate reader (Bio-Rad Laboratories, USA). The rates of proliferation control and PDX1-expressing cells were counted relative to the absorbance on the first day of the experiment. Three independent experiments involving five or six time-point were conducted and this data was averaged.

### 2.5. Migration—Cell Wound Closure Assay

**Figure 1:**
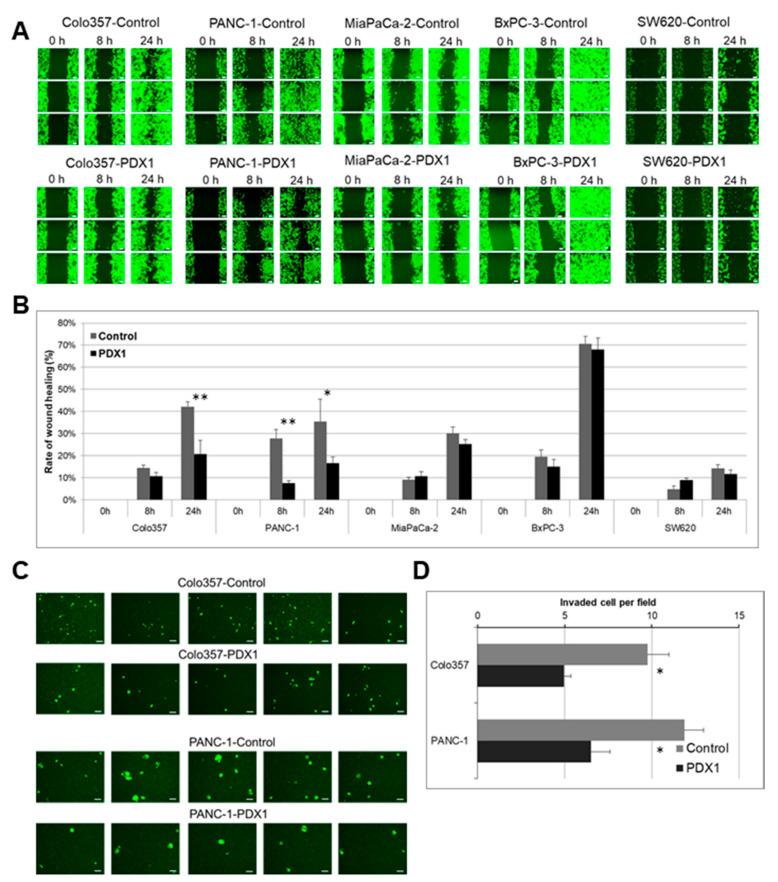
Effect of PDX1 on cell migration and invasion. Colo357, PANC-1, MiaPaCa-2, BxPC-3, and SW620 cell migration (**A**,**B**) analysis and Colo357, PANC-1 cell invasion analysis (**C**,**D**) were performed, as described in “Materials and Methods.” Representative pictures of three independent studies are shown. Magnification ×175. The white stripe corresponds to a scale of 100 µm. Data are presented as the mean ± SD. * *p* < 0.05, ** *p* < 0.01 compared with control group, Manna–Whitney test.

GFP-labeled Control and PDX1-expressing Colo357 and PANC-1 cells (4 ×  10^5^ cells/well) were seeded in 6-well plates to grow in an 80% monolayer for 24 h. Then, a sterile 20–200 μL pipette tip was held vertically to scratch across each well. The detached cells were removed by washing with 1 mL PBS. Two mL of fresh medium was added afterwards and incubated for 8 h and 24 h. Before the image acquisition, the plate was washed with 1 mL of pre-warmed PBS and gently shaken for 30 s. Then, pre-warmed medium or sample was added again and pictures were taken. The scratch closure was monitored and imaged in 0 h, 8 h, and 24 h intervals using a ZOE™ Fluorescent Cell Imager (Bio-Rad Laboratories) at 175× magnification. Three independent experiments for each cell line were conducted, and in each experiment for all lines we analyzed 10–20 fields at 0 h, 8 h, and 24 h. For Figure 1A, three images of each line were chosen. The area of the initial wound was measured, followed by gap area measurements after 8 and 24 h for all images. Control and PDX1-expressing cell migration was assessed by a monolayer gap closure migration assay, using the free ImageJ software (version 1.50i, National Institute of Health, Bethesda, MD, USA).

### 2.6. Transwell Assay

The migration assay was performed in Transwell plates. For the cell migration assay, 2 × 10^5^ cells were seeded on a polycarbonate membrane Transwell inserts containing 8 µm pores (Corning, Corning, NY, USA) and cultured in DMEM without serum. DMEM containing 10% FBS was added to the lower chamber. After incubation for 24 h at 37 °C in a CO2 incubator, the insert was washed with PBS, and cells on the top surface of the insert were removed by a cotton swab. Three independent experiments for each cell line were conducted. GFP-labeled Control and PDX1-expressing Colo357 and PANC-1 cells that migrated to the bottom surface of the insert were counted in 10–20 random fields using a ZOE™ Fluorescent Cell Imager (Bio-Rad Laboratories, USA) at 175× magnification and free ImageJ software (version 1.50i, National Institute of Health, Bethesda, MD, USA). Figure 1C shows five representative images from all experiments. The quantification data are presented in Figure 1D for all images collected in three independent experiments. The error bars represent SD calculated for all replicates.

### 2.7. Motility Gene Expression Measurement

Total RNA was isolated and purified using the RNeasy Mini Kit and subjected to DNAse treatment (Qiagen, Hilden, Germany) in accordance with the manufacturer’s instructions. Following extraction, 5 µg of total RNA was reverse transcribed by Mint Reverse Transcriptase (Evrogen, Moscow, Russia) in accordance with the manufacturer′s instructions. The concentration and quality of RNA and cDNA were determined by spectrophotometry (Nanodrop ND2000c; Thermo Scientific, Waltham, MA, USA). qPCR was performed to determine the gene expression levels in the PDX1-expressing and Control cells on a LightCycler96 Real-Time PCR platform (Roche Applied Science, Mannheim, Germany). The resulting cDNA was mixed with RT^2^ SYBR Green qPCR Mastermix (Qiagen, Valencia, CA, USA) and added to 96-well plates. Gene expression was evaluated using RT^2^ Profiler™ PCR Array Human Cell Motility (Qiagen) plates with immobilized lyophilized primers. The expression levels of four housekeeping genes (*ACTB, B2M, GAPDH, HPRT*) were included in the arrays as a reference and were unchanged under the experimental conditions. The PCR reaction conditions were as follows: 1 cycle at 95 C for 10 min, 45 cycles at 95 °C for 15 s, 60 °C for 60 s; and 1 cycle at 95 °C for 5 s, 55 °C for 60 s and 97 °C for 15 s. Calculations were performed according to [19] for the relative expression ratio.

### 2.8. Quantitative Polymerase Chain Reaction (qPCR) for EMT-Genes Measurement

Total RNA was extracted using Extract RNA reagent (Evrogen, Moscow, Russia) and reverse-transcribed into cDNA using Mint Reverse Transcriptase (Evrogen, Moscow, Russia). qPCR was performed to determine the gene expression levels in the PDX1-expressing and Control cells on a LightCycler480 Real-Time PCR platform (Roche Applied Science, Mannheim, Germany). QPCRmix-HSSYBR was used to determine the relative RNA expression. Primer sequences are shown in Table S1. The *HPRT* and *DDX23* genes were used as an internal control. The PCR reaction conditions were as follows: 1 cycle at 90 °C for 5 min, 40 cycles at 95 °C for 20 s, 60 °C for 20 s and 72 °C for 35 s; and 1 cycle at 95 °C for 5 s, 55 °C for 60 s, and 97 °C for 15 s. The experiments were performed in triplicate for each sample. A relative expression ratio of PDX1 was normalized by using geometric means of the HPRT and DDX23 expression levels. Calculations were performed according to Ganger et al. [19] for the relative expression ratio.

### 2.9. Adhesion

The wells of a 96-well plate were coated with collagen I (Sigma-Aldrich, St. Louis, MO, USA), and fibronectin (Sigma) to assess specific cell adhesion, and poly-L-lysine (Sigma) and bovine serum albumin (BSA, Sigma, USA) to determine non-specific adhesion. 100 μg/mL collagen solutions were used for coating at 40 °C overnight, 10 μg/mL of fibronectin, and 10 μg/mL poly-lysine in PBS were used for coating for 1 h at room temperature. After incubating the plates with the adhesive, the wells were washed with PBS, filled with 3% BSA, incubated for 30 min, and the BSA removed. Next, the same number of cells were scattered into the wells and the plate was placed in a CO2 incubator. After an hour of incubation, the wells were washed three times with PBS to remove non-adherent cells, and the number of adherent cells was assessed using the MTS test.

### 2.10. Statistical Analysis

Data were expressed as the mean (SD) or standard error of the mean (SEM). The significance of differences for the data obtained was estimated using the Wilcoxon–Mann–Whitney U test and the STATISTICA software package (Stat-Soft, Dell Software Company, Round Rock, TX, USA).

## 3. Results

### 3.1. Generation of PDX1-Expressing Cells

**Figure 2:**
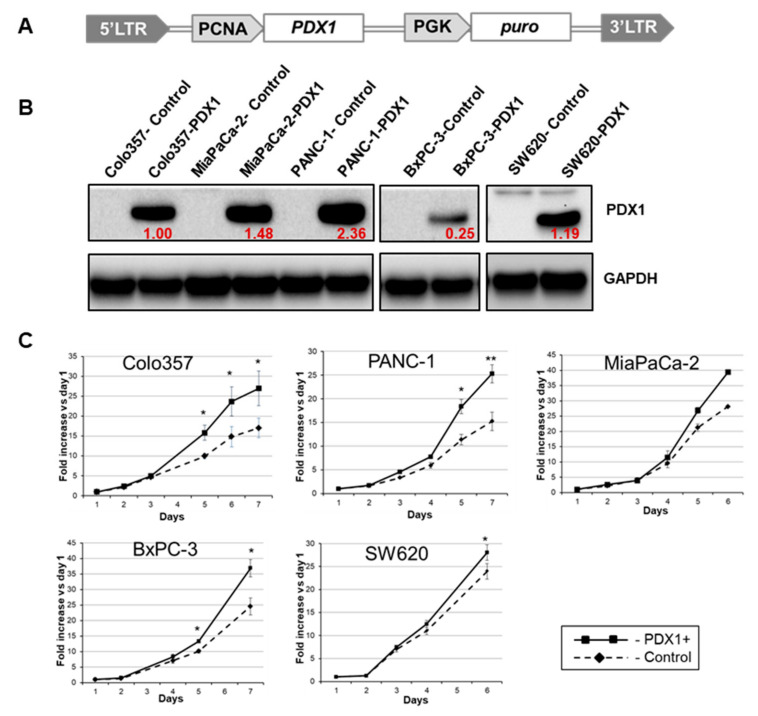
(**A**) Schematic of the lentiviral construct used to generate cultures of pancreatic cancer cells ectopically expressing PDX1. (**B**) Western blot analysis of PDX1 protein content in transduced pancreatic cancer cell lines BxPC-3, Colo357, MiaPaCa-2, PANC-1, and colon cancer line SW620. Red numbers indicate the relative amount of PDX1 protein in transduced Colo357-PDX1, MiaPaCa-2-PDX1, PANC-1-PDX1, BxPC-3-PDX1, and SW620-PDX1 cells. The values are given relative to the level of PDX1 in Colo357-PDX1 cells. GAPDH protein detection in cell extracts was used as a normalizing control. (**C**) Growth kinetics of PDX1-expressing cultures of pancreatic cancer cell lines and corresponding control cells by MTS test performing. Data are normalized relative to values on the first day of the experiment. Three independent experiments involving five–six time-point were conducted. Error bars refer to SD. Cancer cells transduced with lentivirus without the PDX1 gene were used as control. * *p* ≤ 0.05, ** *p* ≤ 0.01.

To investigate the effect of *PDX1* expression on tumor cells in addition to the previously studied PANC-1 line [7], we used pancreatic cancer cell lines with different degrees of EMT: mesenchymal line MiaPaCa-2 and epithelial line BxPC-3. In addition, we included the intermediate phenotype Colo357 cell line in our work as PDAC cells. According to our data, this cell line expresses the ductal differentiation proteins KRT7, KRT19, and SOX9. These cells have mutant K-RAS, which is characteristic of PDAC and rarely found in pancreatic acinar cell carcinoma cells. We included in our experiments colon cancer line SW620 which was selected as closely related to pancreatic cancer cells from the point of view of embryonic origin. Endogenous PDX1 was not detected in native cells. The cells of these lines were transduced with the PDX1-encoding lentivirus described previously [7,20]. A schematic of the lentiviruses obtained is shown in Figure 2A.

Exogenous PDX1 was detected in the protein extracts of cells stably transfected with the *PDX1* gene, and the expression levels differed significantly in different cell lines (Figure 2B). To compare the protein level in PDX1-transduced cancer lines, the relative quantification of PDX1 from protein extracts was assessed. Low levels of PDX1 expression were detected in the most differentiated and least mesenchymal pancreatic cancer cell line, BxPC-3. In the less differentiated, more mesenchymal cell lines Colo357, MiaPaCa-2, and PANC-1, the level of exogenous PDX1 expression was significantly higher (Figure 2B).

### 3.2. Effect of PDX1 Expression on Cell Proliferation

To analyze cell growth kinetics, equal amounts of Colo357, PANC-1, MiaPaCa-2, BxPC 3, and SW620 cells from the control and PDX1 groups were seeded into separate 96-well plates at 1500 and 3000 cells per well. Then, each day for the next 7 days, the metabolic activity of the cells was examined by colorimetric analysis with MTS dye added to the culture medium. Colo357, PANC-1, MiaPaCa-2, BxPC-3, and SW620 cells expressing PDX1 demonstrated an increased proliferation rate compared to control cells (Figure 2C). The greatest difference in the number of cells at the end of the experiment was observed for Colo357 and PANC-1 lines, where the ratio of PDX1 positive cells to control cells was 1.7. The least pronounced effect of *PDX1* expression on proliferative activity was observed in SW620 colon cancer cells.

### 3.3. Influence of PDX1 Expression on Suppression of Pancreatic Cancer Cell Line Migration

We investigated whether the effect of reducing migration properties upon overexpression of the *PDX1* gene on PANC-1 cells previously demonstrated [7,20] was also observed in other pancreatic cancer cell lines and human colon cancer cells. To this end, we performed an in vitro non-directed cancer cell migration study using a wound-healing assay. The cells were additionally labeled with GFP for visualization. Control and PDX1 expressing cells were seeded in equal amounts into the wells of a 6-well plate, a scratch was made the next day, and the wound areas were photographed at the start of the experiment (0 h), and after 8 and 24 h (Figure 1A). Control and PDX1 expressing MiaPaCa-2 and SW620 cells did not exhibit enhanced ability to migrate in vitro and filled the wound area after 8 and 24 h due to proliferation by 13% and 30%, respectively. Control and PDX1-expressing BxPC-3 cells had a high proliferation rate and almost completely filled the wound area by the end of the experiment. However, Colo357 cells expressing PDX1 closed the wound twice as slowly as the control cells (Figure 1A,B). Similarly, the unfilled area of PANC-1 cells expressing PDX1 was also greater after 8 and 24 h of incubation than that of control cells. These data indicate that PDX1 expression can inhibit pancreatic cancer cell motility. Cell invasion analysis was performed for control and PDX1-expressing cancer cells using the Boyden chamber assay (Transwell assay). Cells in serum-free medium were placed in equal amounts in the upper compartment of the Transwell chamber, while the lower compartment was filled with culture medium with standard serum content. After 24 h, cells that had moved to the lower side of the Transwell membrane were photographed and counted (Figure 1C). MiaPaCa-2, BxPC-3, and SW620 cells did not migrate directionally and were not found on the Transwell membrane underside (data not shown). PANC-1 and Colo357 cell lines migrated at once, and their numbers reduced due to the expression of PDX1, as in the non-directed migration assay (Figure 1D).

### 3.4. Effect of PDX1 on the Expression of “Motility” Genes

**Figure 3:**
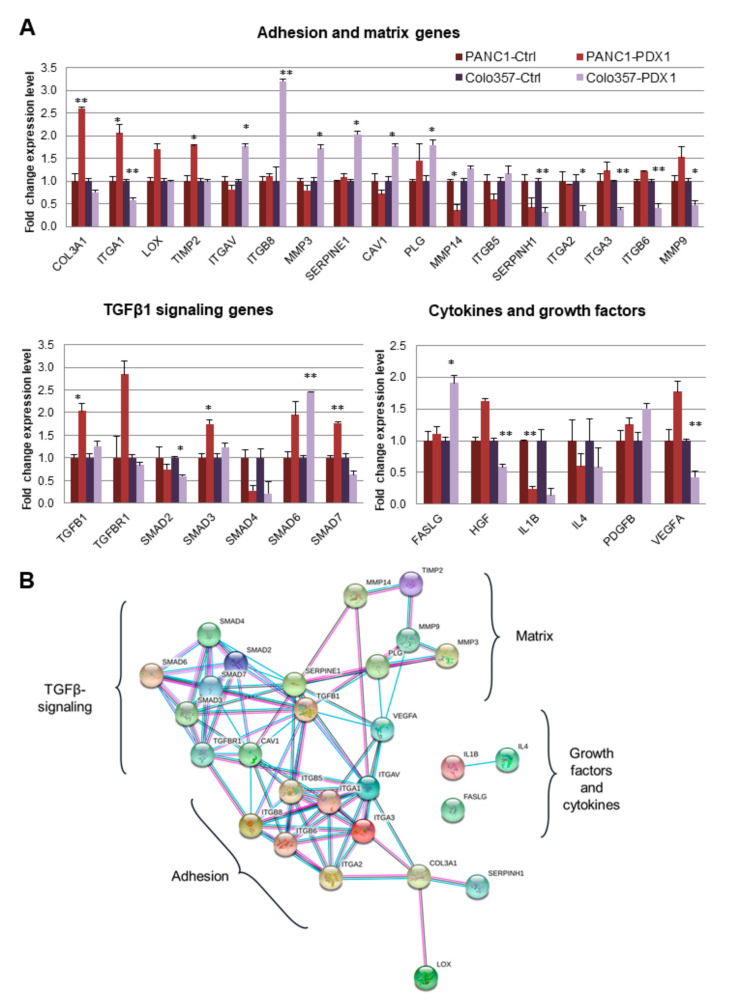
Influence of the PDX1 gene on the motility genes expression. (**A**) Motility genes expression. The data are shown as the mean ± SD (*n* =  3). * *p*  <  0.05, ** *p* < 0.01. (**B**) Functional protein association network of genes with changed expression based on STRING database (https://string-db.org/Version11.0b, Date: 17 October 2020 to 12 August 2021).

To determine the possible causes of reduced cancer cell migration during ectopic expression of PDX1, we analyzed changes in the expression of genes related to cell motility. We used a panel of 86 genes from the RT2 Profiler PCR Array “Cell Motility” (Qiagen). Control and PDX1-expressing cells of Colo357 and PANC-1 lines, with the most pronounced changes in mobility upon expression of PDX1, were analyzed. The results of the analysis are shown in Figure 3A and Figure S1.

The “motility” genes whose transcription levels were altered by PDX1 expression in cells may be divided into three functional groups: (1) genes related to the TGFb-signaling pathway, (2) genes of growth factors and cytokines, and (3) genes of cell adhesion and extracellular matrix remodeling (Figure 3). Changes in expression levels in each of these groups were observed in both cell lines (Figure 3A). Other motility genes in which changes in expression were observed are shown in the heatmaps in Figure S1.

Overexpression of PDX1 in Colo357 cells resulted in decreased expression levels of *ITGA1, ITGA2, ITGA3, ITGB6, MMP9, SERPINH1, SMAD2, SMAD4, HGF, IL4,* and *VEGF* genes. These genes are known to have positive effects on cell motility [21,22,23,24,25,26,27,28,29]. At the same time, the expression levels of *SMAD6, SERPINE1,* and *FASLG*, which negatively regulate cell motility [30,31,32,33,34], increased. A similar effect was observed in PANC-1 cells expressing PDX1. Overexpression of PDX1 in PANC-1 cells led to an increase in the expression levels of *SMAD6, SMAD7, COL3A1, LOX,* and *TIMP2*, which are associated with a decline in cell migration [34,35,36,37,38]. In addition, decreased expression levels of *SERPINH1, MMP14,* and *ITGB5* were associated with elevated cell motility [32,39,40]. Multidirectional effects of PDX1 overexpression in Colo357 and PANC-1 cells were observed for several growth factor genes. These genes can be combined into a common regulatory network. A possible functional protein association network of genes with changed expression based on the STRING database is shown in Figure 3B. Such modeling demonstrates the potential signaling or functional hubs regulated by PDX1 expression.

### 3.5. A Possible TGFβ1-Dependent Mechanism of Reducing Migration

Colo357 and PANC-1 cells are sensitive to TGFβ1 and undergo EMT [41,42,43]. We hypothesized that ectopic expression of PDX1 might affect migration through TGFβ1 factor-dependent mechanisms. We evaluated this hypothesis by comparing the sensitivity of control and PDX1-expressing cells to TGFβ1-induced EMT. Control and PDX1-expressing cells of these lines were seeded into the wells of a 6-well plate, and the next day, the culture medium was changed to serum-depleted medium. After 24 h, the TGFβ1 factor was added at a concentration of 10 ng/mL. Changes in cell phenotype were monitored for 24 and 48 h, after which cells were harvested to analyze changes in the expression level of EMT marker genes.

**Figure 4:**
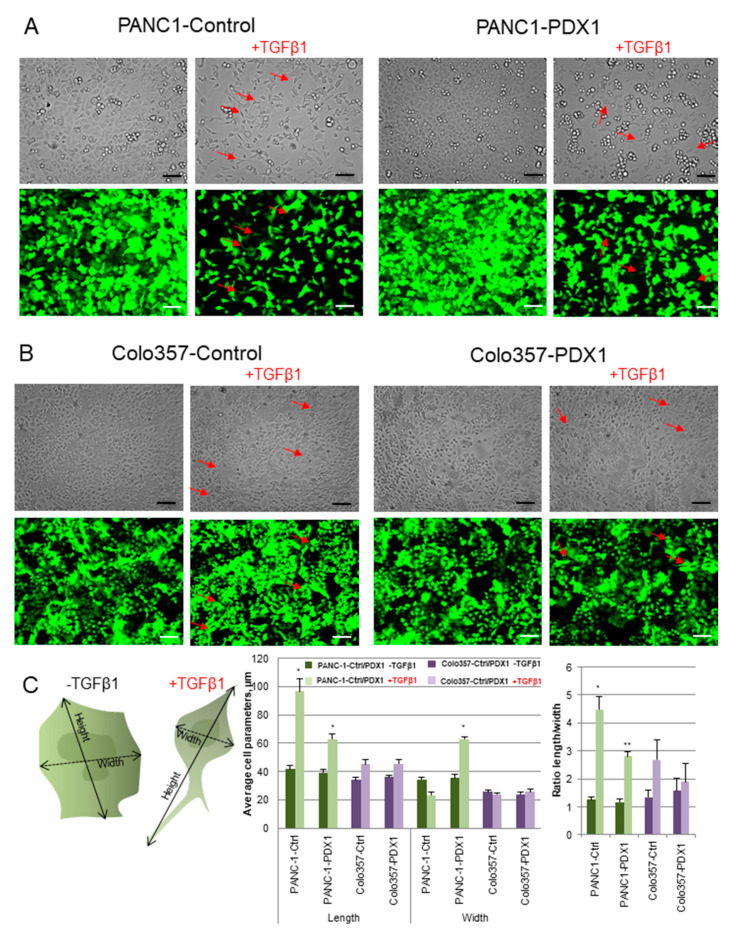
The sensitivity of Control and PDX1-expressing Colo357 and PANC-1 cells to TGFβ1-induced epithelial-mesenchymal transition (EMT). (**A**,**B**) Morphological changes in PANC-1-Control/PDX1 (**A**) and Colo357-Control/PDX1 (**B**) cells after treatment with TGFβ1 factor for 24 h. Magnification ×175. The white and black stripes correspond to a scale of 100 µm. Red arrows indicate examples of fibroblast-like changes. (**C**) Changes in cell parameters cells after treatment with TGFβ1 factor for 24 h: length, width, length-to-width ratio. Data are presented as the mean ± SD. * *p* < 0.05, ** *p* < 0.01 compared with control group, Mann–Whitney test.

After 24 and 48 h of treatment of control Colo357 and PANC-1 cells with TGFβ1 factor, we observed marked changes in cell morphology (Figure 4A,B): cells acquired a fibroblast-like shape characteristic of mesenchymal cells and lost intercellular contacts. At the same time, the cells of both lines expressing PDX1 underwent these changes to a lesser extent: they were less elongated and retained more contacts. To quantify changes in cell morphology, we determined the length and width of cells, and the ratio of length to width before adding and after 24 h of incubation with TGFβ1 (Figure 4C). It was shown that the control PANC-1 cells were lengthened by more than two times after the treatment of cells with TGFβ1. At the same time, PANC-1-PDX1 responded to the addition of TGFβ1 less pronouncedly and extended in length to a lesser extent. Colo357-Control/PDX1 cells were found to be less sensitive to TGFβ1 and showed little change in cell morphology.

**Figure 5:**
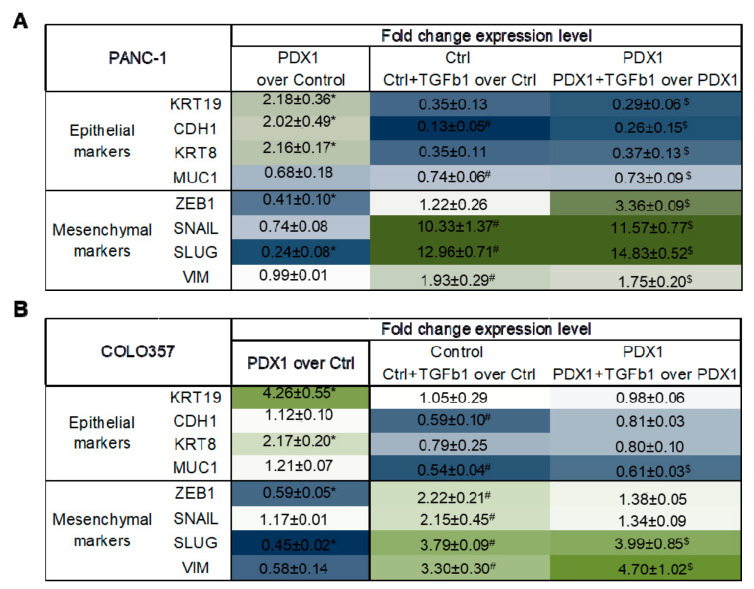
Changes in the expression of EMT marker genes upon treatment of Colo357 with TGFβ1 factor for 48 h. (**A**) Changes in EMT marker gene expression when PANC 1 cells were treated with TGFβ1 factor for 48 h. The data are shown as the mean of relative expression ± SD (*n* = 3). *, ^#^, ^$^ *p* < 0.05 (*- PANC-1-PDX1 over PANC-1-Control, ^#^-PANC-1-Control + TGFb1 over PANC-1-Control, ^$^-PANC-1-PDX1 + TGFb1 over PANC-1-PDX1). (**B**) Changes in the expression of EMT marker genes upon treatment of Colo357 with TGFβ1 factor for 48 h. The data are shown as the mean of relative expression ± SEM (*n* = 3). *, ^#^, ^$^ *p* < 0.05 (*-Colo357-PDX1 over Colo357-Control, ^#^-Colo357-Control + TGFb1 over Colo357-Control, ^$^-Colo357-PDX1 + TGFb1 over Colo357-PDX1). Values of relative expression are highlighted as heatmaps (green-increased expression level, blue-decreased level).

Previously, we showed an increase in gene expression of epithelial markers and a decrease in gene expression of the key EMT regulator, *ZEB1*, in PANC-1 line cells expressing PDX1 [7]. In this study, in PDX1-expressing PANC-1 cells, we confirmed these findings and also revealed increased expression of the epithelial gene *KRT19*, and decreased expression of the EMT regulator gene *SLUG* (Figure 5A). In Colo357 line cells expressing PDX1, an increase in gene expression of epithelial markers *KRT19* and *KRT8*, and a decrease in the expression of mesenchymal markers *VIM*, *SLUG*, and *ZEB1* (Figure 5B) were observed.

One of the key events of EMT is the increase in expression of the EMT regulators *ZEB1* and *SNAIL*, as well as the loss of E-cadherin (*CDH1*) expression. Other changes supporting the hypothesis of TGF-β-related factors in EMT inhibition in *PDX1+* cells are shown in Figure 5.

### 3.6. Reduction of Migration Is Accompanied by Increasing Cell Adhesion

Cell adhesion is associated with the ability of cells to interact with ECM proteins such as collagens of different types, such as laminin and fibronectin (FN). We investigated the adhesion ability of control and PDX1-expressing Colo357 and PANC-1 cells. We coated the wells of a 96-well plate with collagen I and FN to evaluate specific cell adhesion, and poly-L-lysine and BSA to determine nonspecific adhesion.

**Figure 6:**
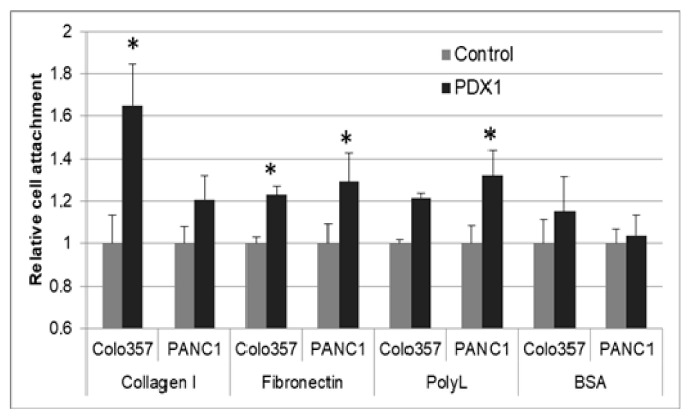
PDX1 gene expression increases cell adhesion. A quantitation of adhesion and cell attachment by cell binding to collagen type I, fibronectin, poly-L-lysine, and BSA (as a control). Data are given as the mean cell attachment ± SEM (*n* = 3). * *p* < 0.05.

Cells expressing PDX1 showed a higher level of adhesion than control cells (Figure 6). The greatest difference in the number of attached cells was observed in Colo357 cells using type I collagen. In addition to an increase in the number of PDX1-expressing Colo357 and PANC-1 cells attached to collagen and FN, increased adhesion to poly-L-lysine was detected.

## 4. Discussion

The invasion and metastasis of malignant tumors is a highly complicated process that depends on multiple steps and factors. Tumor cells must acquire general phenotypic and genotypic changes before they can metastasize. Cell detachment from the primary tumor is considered the initial step of metastasis. The adhesion between cells and the ECM is mediated by cell adhesion molecules. Among them, calcium-dependent receptors, such as cadherins and integrins, play crucial roles [44,45]. Numerous studies have also suggested that epithelial-to-mesenchymal transition (EMT) also contributes to early-stage dissemination of cancer cells and is particularly pivotal for invasion and metastasis of pancreatic ductal adenocarcinoma (PDAC). EMT refers to the biological process by which polar epithelial cells are transformed into mesenchymal cells and is one of the most important mechanisms for malignant tumor cells to acquire the ability of migration and invasion, thus playing a vital role in the occurrence and development of cancer [46,47]. Strengthening of adhesion between tumor cells and ECM of surrounding cells, as well as EMT inhibition, may provide a potential mechanism to inhibit tumor cell migration at the earliest stages. This approach may be especially useful in the case of PDAC, in which early metastasis is characteristic [6].

Recently, we demonstrated that ectopic PDX1 expression resulted in the reduction of PANC-1 cell motility in vitro and in vivo, and here we provide experimental data supporting the hypothesis that suppression of migration may be related to the effect of PDX1 on cell adhesion in primary tumors. To this end, we constructed one colon and four pancreatic cancer cell lines expressing PDX1 ectopically.

### 4.1. “Go or Grow” Hypothesis and PDX1

According to the proliferation assay, pancreatic and colon cancer cells’ ectopic expression of the PDX1 gene increased their growth rate. Directional (Transwell assay) and nondirectional (scratch test) cell migration analyses demonstrated decreased motility of pancreatic Colo357 and PANC-1 cell lines expressing PDX1. Cell motility and the ability of cells to divide are closely related. It is believed that tumor cells cannot divide and migrate at the same time, in a phenomenon called the ‘go or grow’ hypothesis (for a recent review see [48]). The hypothesis states that tumor cells must either temporarily exit the cell cycle in order to invade or invade as a cooperative group of cells, in which some cells continue to proliferate, while others exit the cell cycle to invade and/or migrate, as has been observed in studies of tumor invasive fronts. “Shifting” the number of cells in the direction of more active division should reduce the number of cells capable of migration. A similar increase in the proliferation rate of PDX1-expressing cells is described for the MiaPaCa-2 cell line [49], and PDX1 normally stimulates the division of α- and β-islet cells of the pancreas [15,50]. It was previously shown that PDX1 can stimulate cell proliferation, inhibit apoptosis, and enhance cell invasion [51,52]. PDX1 overexpression stimulates the expression of cyclin D1 and cyclin E and cyclin-dependent kinases CDK2 and CDK4 and suppresses the expression of p21, p27, and p53. This may indicate a role of PDX1 in promoting cell cycle transition from G1 to S stage [52] and simultaneously slowing down cancer cell motility and migration.

Ectopic expression of PDX1 in pancreatic cancer cells showed decreased expression levels of genes associated with increased cell migration capability, as well as increased expression of genes negatively affecting cell motility. In Colo357 cells, ectopic expression of PDX1 decreased the expression levels of integrins *ITGA1*, *ITGA2*, and *ITGA3*, for which a migration-promoting function was shown. For example, suppression of *ITGA1* expression is known to reduce the migration and invasion of hepatocellular carcinoma cells [21] and pancreatic cancer cells [22]. The use of ITGA2 blocking antibodies inhibits migration and induces apoptosis of gastric cancer cells [23,25], while overexpression of ITGA2 stimulates PD-L1 expression by activating the STAT3 signaling cascade, leading to the more aggressive behavior of malignant pancreatic cells [24]. ITGA3 gene knockdown significantly decreases head and neck cancer cell migration and invasion [24], whereas suppression of miR-223 microRNA synthesis, which enhances intracellular signal transmission from ITGA3/ITGB1, stimulates cell motility in prostate cancer [27]. According to our data, the expression level of the ITGB6 gene, whose overexpression is known to facilitate EMT and is associated with the invasiveness of oral cancer cells, decreases in cells expressing PDX1 [28]. The migration test results obtained here agreed with the previous data.

Data contradicting the “go or grow” hypothesis were also found in our study. Thus, in cells expressing PDX1, there was an increase in the expression level of the gene 5 alpha subunit of integrin (integrin subunit alpha V), reported to stimulate proliferation, migration, and invasion of gastric cancer cells [30], and an increase in the expression level of integrin subunit beta 8, a positive regulator of cell migration, since ITGB8 knockdown leads to a reduction in cell motility [31]. In Colo357 cells expressing PDX1, there is an increase in MMP3 gene expression, and high expression levels in oral squamous cell carcinoma cells are associated with metastasis and poor prognosis for patients [53]. Pancreatic cancer cell lines ectopically expressing PDX1 in our study had a decreased level of SERPINH1, a positive regulator of cell motility. Suppression of SERPINH1 in gastric cancer cells is known to reduce survival, colony formation, migration, and invasion, whereas overexpression enhances these traits [29].

### 4.2. Epithelial-Mesenchymal Transition and PDX1

The analysis of “motility” gene expression revealed changes in the transcription of genes associated with the transforming growth factor-β (TGFβ)-signaling pathway. TGF-β is a secreted multifunctional cytokine that strongly regulates the activity of immune cells while, in parallel, can promote malignant features such as cancer cell invasion and migration, angiogenesis, and the emergence of cancer-associated fibroblasts. TGF-β is abundantly expressed in cancers, and most often, its abundance is associated with poor clinical outcomes. A biphasic role of TGF-β has been proposed in cancer progression. In the early stages of oncogenesis, it acts as a tumor suppressor by mediating growth arrest; however, it can act as a tumor promoter in advanced stages by supporting invasion and metastasis of cancer cells, while its cytostatic effects are blocked by the rewiring of its signaling during malignant transformation [54,55].

Modulation of the adhesive properties of cancer cells also occurs during the initiation of the EMT process. A direct correlation between EMT and cell invasion has also been reported [56]. EMT in cancer cells can be induced by the activation of various internal and external signaling pathways. TGFβ is one of the most important inducers of EMT [57]. When studying the expression of motility genes, we found changes in the expression of genes associated with the TGF-β signaling pathway in pancreatic cancer cells with ectopic expression of PDX1. Decreased expression of *SMAD2* and *SMAD4* genes, central proteins in the signal transduction pathway of TGFβ1 which triggers EMT, were observed. miR-19a-3p, which targets the downstream effectors of TGFβ signaling, SMAD2, and SMAD4, is known to inhibit prostate cancer cell invasion and migration [58]. It also increases the expression levels of SMAD6 and SMAD7 genes that inhibit TGF-β signaling and EMT activation. Suppression of SMAD6 expression promotes endothelial cell proliferation and migration through the activation of BMP signaling [59].

Based on the data obtained, we hypothesized that since ectopic expression of PDX1 in pancreatic cancer cells decreased the expression of positive regulators and increased the expression of negative regulators of TGFβ signaling, the addition of TGFβ1 to the medium would also alter the cellular response. According to our data, ectopic expression of PDX1 decreased cell sensitivity to TGFβ1. The expression of the epithelial marker gene, E-cadherin, was mildly reduced, and the expression of the EMT regulator genes, ZEB1 and SNAIL, were mildly induced. In addition, when stimulated with TGFβ1, phenotypic changes in pancreatic cancer cells were inconspicuous, and control cells were more likely to acquire the fibroblast-like shape characteristic of cells undergoing EMT, lose intercellular contacts, and become more isolated from each other.

### 4.3. Pancreatic Cancer Cell Adhesion to Extracellular Matrix and PDX1

Interaction between cancer cells and ECM proteins influences cell migration and invasion. PDX1-expressing cells of the Colo357 and PANC-1 lines demonstrated a higher level of adhesion to collagen type I, FN, and poly-L-lysine than control cells. For collagen type I, it is known that the formation of soft collagen gels reduces migration and promotes the formation of a non-invasive cell phenotype, and the formation of stiffer collagen fibers, which are more adhesive in nature, leads to a change to more adhesive cells and limits their migration [60]. The observed increase in the adhesion of cancer cells to FN may also play an important role in migration and invasion. The absence of FN synthesis by malignant cells gives them an advantage when migrating within tissues [61]. On the other hand, the ability to interact with the FN matrix of other cells may be important for cell invasion. To initiate metastasis, tumor cells must interact with the capillary endothelium and penetrate the surrounding tissues. The affinity of metastatic cells to the ECM of endothelial cells has been reported [62]. FN seems to play a role, as anti-FN antibodies can partially prevent this adhesion. It has been shown that the interaction of tumor cells with FN-rich fibers is sufficient to increase cell migration [63]. The increased adhesion of pancreatic cancer cells expressing PDX1 to the cationic polypeptide poly-L-lysine detected also indicates a decrease in the migration potential of cancer cells. It is known that the use of hyperadhesive substrates coated with polylysine inhibits cancer cell motility [64].

In summary, the ectopic expression of PDX1 reduces the migration potential of cancer cells at early stages, particularly by increasing the adhesive properties of cells and reducing the sensitivity of cells to TGFβ1-induced EMT.

## 5. Conclusions

Despite decades of study, metastasis remains the major cause of mortality in patients with cancer. This is the most complex and deadly event in cancer progression. Metastasis of cancer cells accounts for 90% of deaths from solid tumors, however, because efficient anti-invasion and anti-metastatic drugs are still lacking. The initiation of metastasis relies on the tumor cell cross-talking with stromal cells and involves cell-cell adhesion changes. The influence of many oncogenic signaling pathways leads to, in particular, epithelial-mesenchymal transition (EMT) in single cancer cells, and a hybrid EMT in collective migratory cells [65].

The adhesion molecules, particularly E-cadherin, connected cytoskeletal components, and cytoskeleton-binding proteins, provide novel promising targets in metastatic cancer treatment [66]. However, the involvement of E-cadherin in metastasis is complex; local tissue invasion is not the rate-limiting step of metastasis, and the earlier assumption about the loss of E-cadherin resulting in invasion and thereby, in metastasis, is an oversimplification. The observation that adherent clusters of circulating tumor cells were more metastatic than individual circulating tumor cells, and that many metastases expressed high levels of E-cadherin, further underscores the complexity of the mechanisms of metastasis. Perhaps inhibiting E-cadherin, rather than activating it, could suppress cancer metastasis [67,68].

In addition, traditional views of metastasis assume that it occurs late during evolution. We now know, however, that metastasis often occurs prior to clinical detection of primary tumors [65,69].

Despite real progress in the field, which has resulted in the identification of key drivers of the metastatic steps, research in this area is still ongoing, to shed more light on the cross-communication occurring between contributing factors in each step [65].

Here, we demonstrate the potential metastatic inhibitory functions for PDX1, a transcription factor indispensable for pancreatic development. According to our observations, PDX1 ectopic expression of PDX1 reduced the migration potential of cancer cells, in particular, by increasing the adhesive properties of cells and reducing the sensitivity of cells to TGFβ1-induced EMT. It is known that upon neoplastic transformation, the role of PDX1 changes from tumor-suppressive to oncogenic [15]. These distinct roles of PDX1 at different stages suggest that therapeutic applications targeting PDX1 need to correspond to its changing functions at different stages of carcinogenesis.

Our study highlights a potential narrow window for the therapeutic application of PDX1 as an antimetastatic agent in the initial stages of cancer development in patients that are without early metastasis.

Future studies are needed to identify more precisely how PDX1 may interact with divergent sets of coregulators to modulate critical aspects of metastasis and provide important insights into specific disease vulnerabilities that can be targeted for personalized therapeutic intervention. In particular, such future studies would include modeling a potential PDX1 gene-therapeutic effect by delivering genes encoding PDX-1 into cells as part of an expressing construct both in cell cultures and in animal models.

## Data Availability

The data presented in this study are available on request from the corresponding author.

## Supplementary Materials

The following are available online at https://www.mdpi.com/article/10.3390/cancers13174390/s1, Table S1: Real-time PCR primers used in gene expression analysis, Figure S1. The heatmap of relative motility genes expression.
