# Supplementary material for: Pancreatic Lineage Specifier PDX1 Increases Adhesion and Decreases Motility of Cancer Cells
Source: Cancers (Basel). 2021 Aug 30;13(17):4390. doi: 10.3390/cancers13174390 (PMC8430990; doi:10.3390/cancers13174390)
Supplement: Supplementary file 1 [file cancers-13-04390-s001.zip › cancers-1316583-supplementary.pdf]

Table S1. Real-time PCR primers used in gene expression analysis.

Gene		Primer Sequence (5′ → 3′)	PCR Product (bp)
HPRT	Fw	GCTATAAATTCTTTGCTGACCTGCTG	140
	Rv	AATTACTTTTATGTCCCCTGTTGACTGG	
DDX23	Fw	TATACAGCGTCAGGCAATTC	147
	Rv	GACTCTTCGATCCTGTCAAT	
KRT19	Fw	CTGGGCTTCAATACCGCTGA	170
	Rv	GCAGGTCCGAGGTTACTGAC	
CDH1	Fw	AGTGCCTGCTTTTGATGATG	338
	Rv	AGCTTGAACTGCCGAAAAATC	
KRT8	Fw	ATGTTGTCCATGTTGCTTCG	125
	Rv	ACCCTCAACAAGTTTGCC	
MUC1	Fw	CTGGTCTGTGTTCTGGTTGC	250
	Rv	CCACTGCTGGGTTTGTGTAAG	
VIM	Fw	GCAGAAGAATGGTACAAATCCA	144
	Rv	TTTAAGGGCATCCACTTCACA	
SNAIL	Fw	CCAATCGGAAGCCTAACTAC	124
	Rv	GCGGTGGGGTTGAGGATCTC	
SLUG	Fw	AGAAGGTTTTGGAGCAGTTTTTG	160
	Rv	TGGTTGCTTCAAGGACACAT	
ZEB1	Fw	GAACAGTGTTCCATGCTTAAGAGCG	217
	Rv	GGGCGGTGTAGAATCAGAGTCATTC	

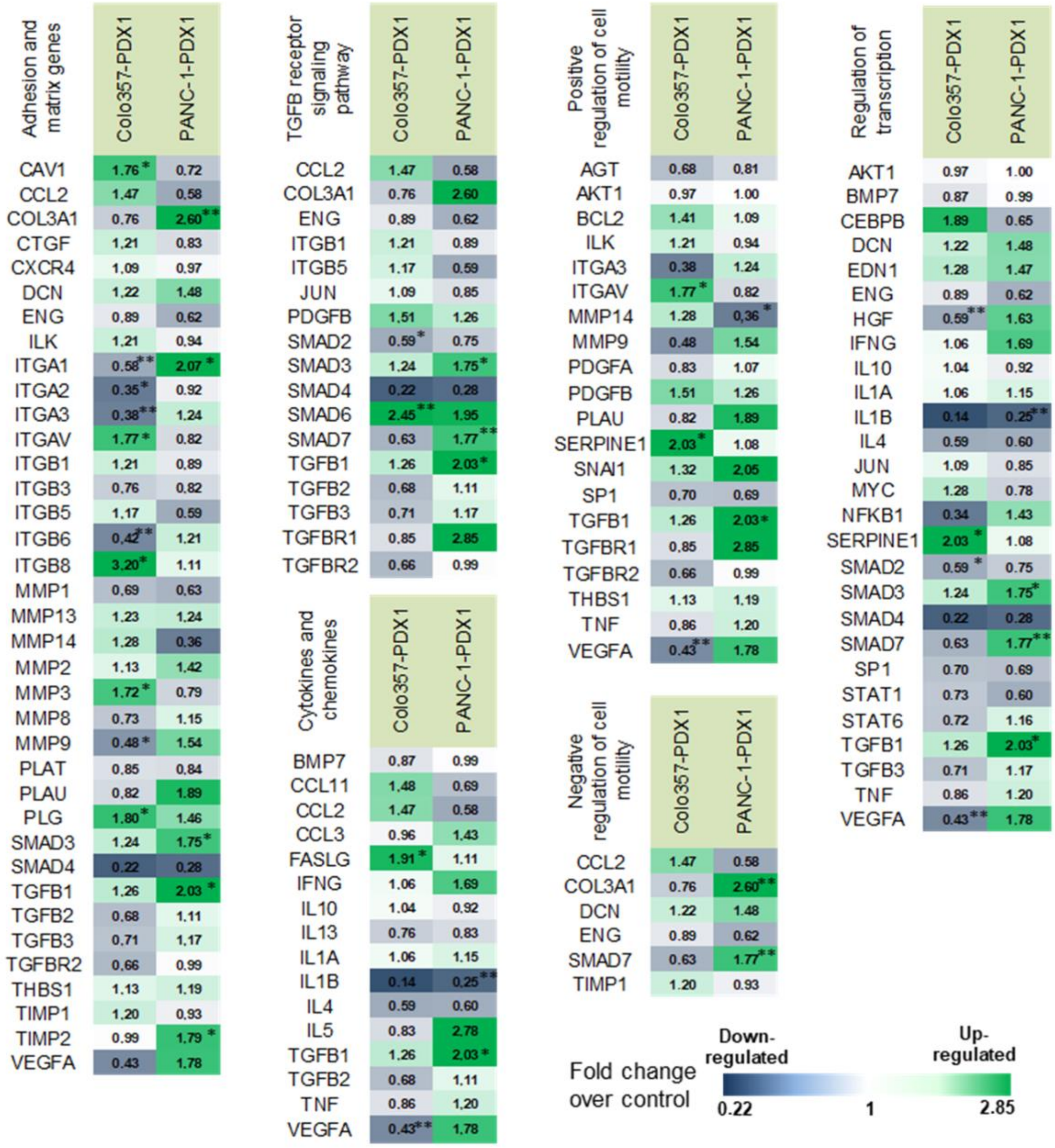

Figure S1. The heatmap of relative motility genes expression. The fold change expression of 5 groups of genes from the RT2 Profiler PCR Array "Cell Motility" in Colo357-PDX1 over Colo357-Control and PANC-1-PDX1 over PANC-1-Control cells. Green - increased expression level over control, blue - decreased level. The data are shown as the mean of relative expression (n=3). *$p < 0.05$, **$p<0.01$, Manna-Whitney test.
